# Effects of Letrozole-HMG and Clomiphene-HMG on Incidence of Luteinized Unruptured Follicle Syndrome in Infertile Women Undergoing Induction Ovulation and Intrauterine Insemination: A Randomised Trial

**DOI:** 10.5539/gjhs.v8n4p244

**Published:** 2015-08-31

**Authors:** Azra Azmoodeh, Mansoureh Pejman Manesh, Firouzeh Akbari Asbagh, Azizeh Ghaseminejad, Zeinab Hamzehgardeshi

**Affiliations:** 1 Department of Reproduction & Infertility, Mirza Kouchak Khan Women’s Hospital, Tehran University of Medical Sciences, Tehran, Iran; 2 Department of Midwifery and Reproductive Health, Mazandaran University of Medical Sciences, Sari, Iran; 3 Traditional and Complementary Medicine Research Centre, Mazandaran University of Medical Sciences, Sari, Iran

**Keywords:** letrozole, clomiphene, luteinized unruptured follicle syndrome, induction ovulation, intrauterine insemination

## Abstract

**Background:**

Luteinized unruptured follicle (LUF) syndrome is considered a cause of ovulation failure and a subtle cause of infertility. Preovulatory injection of human chorionic gonadotropin (HCG) prevents or treats LUF syndrome, but it has also occurred after the induction of ovulation with clomiphene/HMG and HCG. This study was designed for evaluation and comparison of LUF incidence in eligible infertile women undergoing two stimulation protocols (clomiphene + HMG and letrozole + HMG) in addition to intrauterine insemination (IUI). Some related factors were compared between LUF and non-LUF cycles as secondary outcomes.

**Methods:**

The study was designed as a prospective randomized controlled trial. Patients were randomized using a table of random numbers into two equal protocol groups. For group A, (n = 90) clomiphene citrate was administrated orally in doses of 100 mg/day, and group B (n = 90) orally received letrozole 5 mg/day from day 3 to 7 of the menstrual cycle. Then HMG 75IU/day was administered intramuscularly in both groups on day 8 of the menstrual cycle and the dose was adjusted on the basis of ovarian response. The optimum size of preovulatory follicles for the injection of HCG (10,000 IU) was considered 18–23 mm. The number and size of preovulatory follicles were assessed by vaginal ultrasound 12 h before HCG (D0). Endometrial thickness was measured as well. IUI was performed on all patients 38–40 h after HCG. The second ultrasound examination was performed to observe the evidence of oocyte releasing at the time of IUI (D1). If the follicles were unruptured, a third sonography was performed on day 7 after HCG (D7) to observe LUF syndrome.

**Results:**

There was a significant difference between clomiphene-HMG and letrozole-HMG in LUF (p = 0.021) and pregnancy (p = 0.041). The complete LUF in letrozole-HMG was lower than the alternative group and the pregnancy rate was higher. The patients in the non-LUF group had higher midluteal progesterone and a thicker endometrium compared to LUF cycles (p = 0.039) and (p < 0.001). The results of our multivariate logistic regression indicate that size 18–19.9 mm leads to the complete LUF less than ≥22 mm [AOR: 0.25, P = 0.005], and in size 20– 21.9 mm as well [AOR: 0.17, P = 0.002].

**Conclusion:**

Letrozole, with lower incidences of LUF, is more effective than clomiphene citrate for the induction of ovulation in IUI cycles. In our study, we illustrated that larger follicles of ≥22 mm diameter were associated with higher incidences of LUF. We recommend that further studies investigate and focus on the relationship between follicular size and/or full hormonal profiles and LUF.

## 1. Introduction

Luteinized unruptured follicle (LUF) syndrome is considered to be a cause of ovulation failure and a subtle cause of infertility, that despite an unruptured follicle, luteinization occurs because of action the Luteinizing Hormones (LH) (Qublan et al., 2006). LUF is observed in 10% of natural menstrual cycles in fertile women, but in stimulated cycles it is higher (1). The incidence of LUF has been reported to be 25–43% in infertile women, and the recurrent rate in 2–3 consecutive cycles is 78.6% and 90% respectively (Wang, Qiao, Liu, & Lian, 2008).

Many conditions can be attributed to LUF occurrence, such as unexplained infertility, endometriosis, pelvic adhesions, and non-steroidal anti-inflammatory drugs (NSAID), hyperprolactinemia, a defect in LH surge, or abnormal Follicle Stimulating Hormones (FSH) (Qublan et al., 2006). LUF cycles consist of non-conception (Qublan et al., 2006) and the pregnancy rate after intrauterine insemination (IUI), which is performed regardless of the evidence of follicular rupture, decreased as a result of LUF (M. E. Ghanem et al., 2011). It has been suggested that preovulatory injection of human chorionic gonadotropin (HCG) prevents or treats LUF syndrome, but it has also occurred after the induction of ovulation with clomiphene/HMG and HCG (Coetsier & Dhont, 1996).

Some studies have suggested a high incidence of LUF in consecutive cycles with clomiphene in unexplained infertile women and in PCOs patients compared to cc/HMG and HMG alone (M. E. S. Ghanem et al., 2009; Qublan et al., 2006). Letrozole had a lower LUF incidence than clomiphene citrate only in one investigation (Wang, Lü, Cao, & Li, 2008). Some researchers have proposed other options for treatment of these types of patients, such as IVF, which may be the only effective treatment for LUF patients (Sonntag & Ludwig, 2012). However, it is cost-ineffective and the procedure is invasive. Letrozole is an aromatase inhibitor that stimulates FSH release by blocking the conversion of androgen to estradiol and increases FSH receptors in the ovarian tissue. Letrozole is a suitable and cost-effective ovarian stimulation agent that is associated with higher ovulation and pregnancy rates compared to clomiphene (M. R. Begum, J. Ferdous, A. Begum, & E. Quadir, 2009; Elsedeek & Elmaghraby, 2011; Ganesh, Goswami, Chattopadhyay, Chaudhury, & Chakravarty, 2009).

According to a study that researched the possibility of interference of clomiphene citrate on the etiology of LUF (Qublan et al., 2006), so far few studies have investigated the incidence of LUF in other ovarian stimulation regimens such as Letrozole.

### 1.1 Objectives

This study was designed to evaluate and compare the incidence rate of LUF in eligible infertile women undergoing two stimulation protocols: clomiphene + HMG and Letrozole + HMG, and IUI. The secondary aim of this study was to evaluate other factors such as dominant follicle size, hormonal profiles (luteal phase progesterone, FSH, and LH), and endometrial thickness in LUF and non-LUF cycles.

## 2. Methods

This study was conducted in the infertility and IVF ward of Hospital University in 2014. The study was designed as a prospective randomized controlled trial. This study was a randomized, single blind clinical trial that was registered with a number IRCT2014091019113N1. It was determined that a sample size of 196 subjects was needed to compare the two groups. The confidence level was 95%. One hundred and eighty subjects were analyzed.

All of the patients who met the inclusion criteria were counseled and granted informed consent before entrance. A routine primary evaluation, such as seminal fluid analysis, hysterosalpingogram (HSG), thyroid function test (TSH), serum prolactin concentration, baseline FSH, LH levels, and vaginal sonography (on the second or third day of their cycles) was performed.

### 2.1 Participants

**Inclusion criteria**

Inclusion criteria included infertile women aged 18–35 years old, (who failed to conceive after 12 months of regular intercourse without contraception). The participants were candidates for mild controlled ovarian stimulation and IUI at the first cycle.

**Exclusion criteria**

Exclusion criteria included hyperprolactinemia, thyroid dysfunction, ovarian cyst detected in baseline ultrasound, endometriosis, patients who took an NSAID, tubal or uterine problems detected by HSG, and an abnormal FSH (>10 mIU/ml).

**Trial design and randomisation: sequence generation**

Patients were randomized using a table of random numbers into two equal protocol groups.

**Study settings**

Mirza Kouchak Khan Women’s Hospital, Tehran University of Medical Sciences, Tehran, Iran

**Interventions**

Protocol groups included group A, and group B. For group A, clomiphene citrate (Clomid^®^, Aventis, Ohio, USA) was administrated orally in doses of 100 mg/day. Group B orally received letrozole (Femara^®^, Novartis New York, NY, USA) 5 mg/day from day 3 to 7 of the menstrual cycle. Then HMG 75IU/day (Merional^®^, IBSA, Lugano, Switzerland) was administered intramuscularly to both groups on day 8 of the menstrual cycle and the dose was adjusted based on ovarian response. Transvaginal sonography (Simens G40, Germany, Vaginal probe 5 Mz) was performed on day 10 or 11 of the menstrual cycle in order to monitor follicular growth. Transvaginal ultrasounds were then conducted every other day. The size of the follicles was measured in the inner transverse and longitudinal planes, and then the mean diameter was calculated. The optimum size of preovulatory follicles for an injection of HCG was considered 18–23 mm. When at least one follicle reached a mean size of 18 mm or more, 10,000 IU HCG (Choriomon^®^, IBSA, Lugano, Switzerland) was administered intramuscularly to trigger ovulation. The number and size of preovulatory follicles were assessed by vaginal ultrasound 12 h before HCG (D0). Endometrial thickness was measured as well (at the greatest diameter vertical to the midsagittal plane near the fundus). IUI was performed on all patients, 38–40 h after HCG, and the second ultrasound examination was performed to observe evidence of oocyte releasing at the time of IUI (D1). If the follicles were unruptured, then the third sonography was performed on day 7 after HCG (D7) to observe LUF syndrome. For all patients, midluteal progesterone was assessed on D7.

Ovulation was determined based on the complete collapse of the preovulatory follicle or the shrinkage of it to at least 50% of its primary size with or without free fluid in the pouch of Douglas and serum midluteal progesterone ≥10 ng/ml. LUF was diagnosed when none of the follicles ruptured and midluteal P level >3 ng/ml. Serum βhCG was measured 1 week after the missed period in order to confirm a biochemical pregnancy. Luteal phase support by progesterone was given to none of the patients. All hormonal assays were conducted by ELISA. If during intervention, hyperstimulation occurred, the cycle was canceled and the patients were excluded from the study.

**Outcomes**

Primary outcome: Incidence of LUF between the two medication groups.

Secondary outcome: Other factors such as dominant follicle size, hormonal profiles (luteal phase progesterone, FSH, and LH), and endometrial thickness in LUF and non-LUF cycles.

### 2.2 Sample Size


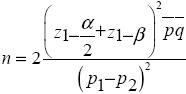


*Alfa*=0.05

*Z_1-a/2_*=1.96

*Beta*=0.2

*Z_1-B_*=0.84

*p1*=0.4

*p=inline*=0.3

*p2*=0.2

*n*=83

### 2.3 Blinding

Single blind clinical trial.

### 2.4 Statistical Methods

Continuous variables were described by mean and standard deviation, and categorical variables were described by frequency and percent. Univariate analysis, evaluating homogeneity, and comparing two groups of intervention were performed using the chi-square test, Fisher’s exact test, an independent t-test, and the Mann–Whitney U test. Multivariate models were conducted by using a logistic regression model by entering the significant variables in the univariate analysis. The p value of <0.05 was considered significant. All analysis was performed using SPSS, version 16 for Windows.

### 2.5 Ethics

Ethical approval was obtained from the Ethics Committee of the Tehran University of Medical Sciences & Informed consent was obtained from all patients involved in the study.

**Figure:**
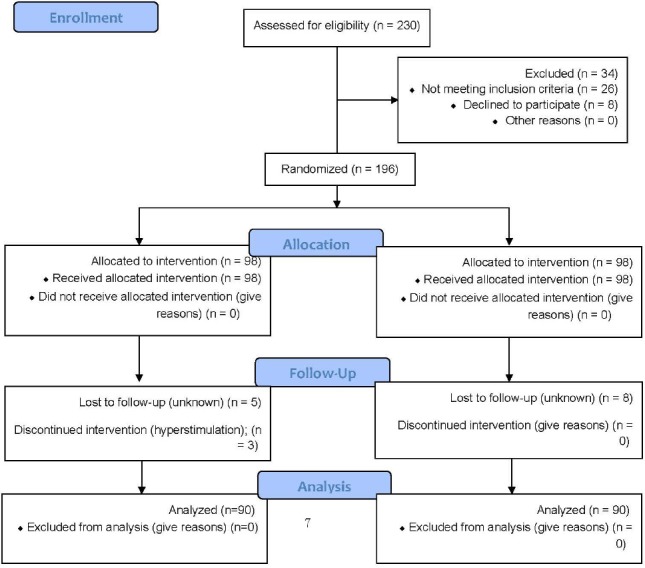
CONSORT 2010 Flow Diagram

## 3. Results

### 3.1 Participant Flow

As shown in the CONSORT flow-chart, there were 196 randomized patients in this study, with 98 patients in each group. Five cases were lost in the CC-HMG group, and for three cases intervention was discontinued because of hyperstimulation. In the letrozol-HMG group, eight cases were lost. In total, 180 females were included in this study (90 patients in each group).

### 3.2 Baseline Data

The mean age was 28.47±4.2, and the ages ranged from 18 to 35 years old. The mean duration of infertility (in years) was 2.91±1.83 (ranged 1–9 years). The highest cause of infertility was unexplained infertility (41.1%), followed by PCO (34.4%). The lowest cause of infertility was male factor (24.4%).

Two groups (clomiphene-HMG and letrozole-HMG) were homogenous in age, duration of infertility, FSH, LH, cause, and type of infertility (Table 1).

**Table 1:** Characteristics of patients undergoing ovulation induction with letrozole and clomiphene citrate (CC)

Variable	Clomiphene-HMG	Letrozole-HMG	p-value
Age (mean ± sd); year	28.13 ± 4.2	28.6 ± 4.4	0.345
Duration of infertility(mean ± sd); year	2.94 ± 1.8	2.87 ± 1.9	0.749
FSH (mean ± sd); mIU/ml	6.24 ± 1.3	6.22 ± 1.5	0.649
LH (mean ± sd); mIU/ml	6.96 ± 2.8	6.8 ± 3.3	0.392
**Cause, n (%)**			
PCO	31 (34.4)	31 (34.4)	0.314
Unexplained infertility	33 (36.7)	41 (45.6)	
Male factor	26 (29)	18 (20)	
**Type of infertility**			
Primary	55 (62.2)	61 (67.8)	0.342
Secondary	33 (37.8)	29 (32.2)	

### 3.3 Outcomes and Estimation

The primary outcome was the incidence of LUF between the two medication groups. There was a significant difference between clomiphene-HMG and letrozole-HMG in LUF (p = 0.021) and pregnancy (p = 0.041). The complete LUF in letrozole-HMG was lower, and also the pregnancy rate was higher than the alternative group. There were no significant differences between the two groups in other variables (Table 2).

**Table 2:** Comparing progesterone, endometrial thickness, pregnancy, LUF, and size of dominant follicle between the two groups of study

Variable	Clomiphene-HMG	Letrozole-HMG	p-value
Progesterone (mean ± sd); ng/ml	22.15 ± 12.2	22.06 ± 13.0	0.649
Endometrial thickness(mean ± sd); mm	7.49 ± 1.6	7.9 ± 1.5	0.069
**Pregnancy, n (%)**			
No	66 (73.3)	53 (58.9)	0.041
Yes	24 (26.7)	37 (41.1)	
**Cycle**			
Ovulatory	58 (64.4)	73 (81.1)	0.012
Complete LUF	32 (35.6)	17 (18.9)	
**Size (mm), n (%)**			
18–19.9	42 (46.7)	33 (36.7)	
20–21.9	37 (41.1)	45 (50)	0.386
≥22	11 (12.2)	12 (13.3)	

To evaluate the correlation of other factors and LUF, such as basal FSH and LH, endometrial thickness, midluteal progesterone, cause of infertility, and the size of the largest preovulatory follicles, the comparison was accomplished between LUF and non-LUF cycles.

As shown in Table 3, the patients in the non-LUF group had a significant higher midluteal progesterone (p = 0.039) and a longer luteal phase duration compared with complete LUF (p < 0.001). Endometrial thickness was significantly thicker in non-LUF patients (p < 0.001). Mean basal serum FSH in complete LUF cycles had a significantly higher than the non-LUF group (p = 0.046). The pregnancy rate in the LUF group was 0%. Complete LUF occurred in patients with unexplained infertility more than those with a male factor (p = 0.005). There was no difference in the occurrence of complete LUF between PCO and unexplained infertility and no difference between PCO and a male factor (Table 3).

**Table 3:** Univariate analysis comparing complete LUF with non-LUF in different variables

Variable	Completed LUF	Non-LUF	OR	p-value
Age (mean ± sd); year	28.2 ±4.3	28.43 ± 14.34	0.99	0.757
Progesterone (mean ± sd)	18.91 ± 10.8	23.31 ± 13	0.97	0.039
Luteal phase (mean ± sd); day	13.10 ± 1.81	16.31 ± 12.53	0.57	<0.001
Duration of infertility (mean ± sd); year	2.77 ± 1.74	2.96 ± 1.88	0.94	0.523
LH (mean ± sd); mIU/ml	7.23 ± 3.79	6.78 ± 2.73	1.05	0.379
Endometrial thickness (mean±sd); mm	6.67 ± 1.06	8.11 ± 1.55	0.428	<0.001
Pregnancy, n (%)	0	61(47)	0	0.997
**Cause, n (%)**				
PCO	17(34.7)	45(34.4)	2.95	0.051
Unexplained infertility	27(55)	47(36)	4.48	0.005
Male factor	5(10.2)	39(30)	Reference	Reference
**Size (mm), n (%)**				
18–19.9	21(28)	54(72)	0.42	0.08
20–21.9	17(21)	65(79)	0.29	0.01
≥22	11(48)	12(52)	1 (ref.)	1 (ref.)

There was no significant relationship between diagnosis (complete LUF and non- LUF) and other variables.

To compare the two protocols in the study the effect of the significant variables in the univariate analysis were adjusted. Table 4 shows the result of the multivariate analysis. With adjusting the effect of FSH, and the cause, the two protocols compared with the logistic model. Like the univariate analysis, complete LUF in the letrozole-HMG protocol had a significantly lower occurrence than the alternative protocol (p = 0.007). In addition, the results of our multivariate logistic regression indicate that the odds of complete LUF in size 18–19.9 mm is lower than ≥22 mm [AOR: 0.25, P = 0.015], and in size 20–21.9 mm is [AOR: 0.17, P = 0.002], as well (Table 4).

**Table 4:** Logistic regression model comparing diagnostics with two protocols

Variable	OR	p-value
**Size (mm)**		
18–19.9	0.25	0.015
20–21.9	0.17	0.002
≥22	Reference	Reference
FSH	1.37	0.017
Protocol, clomiphene-HMG	3.15	0.003
**Cause**		
PCO	3.9	0.021
Unexplained infertility	5.77	0.002
Male factor	Reference	Reference

## 4. Discussion

The current study’s results showed that complete LUF was lower in the letrozole-HMG group, and that the pregnancy rate was higher than the alternative group. The size of the preovulatory follicles during the injection of HCG was an important predictor of complete LUF.

The current study is similar to the other study that showed that LUF was lower in the letrozole group compared to the clomiphene group. This study compared the incidence of LUF between clomiphene and letrozole in polycystic ovarian syndrome (PCOS) patients, and showed the lower rate of LUF in the letrozole group (p < 0.05) (Wang, Lü et al., 2008).

In addition, in an investigation of Gublan-H et al. (2006), LUF in unexplained infertility patients who were administrated CC-HMG during the first cycle occurred at a rate of 25.1%, with a higher incidence in consecutive cycles (56.5% and 58.9% in the second and third cycles, respectively) and a recurrence rate of 78.6% and 90%, respectively (Qublan et al., 2006).

Ghanem et al. (2009) investigated the “Effect of stimulation protocol on the risk of LUF in polycystic ovarian syndrome” and found that the incidence of LUF was higher in CC cycles versus CC-HMG and HMG alone (15.1%, 6.9%, and 5.2% respectively) (M. E. S. Ghanem et al., 2009).

Some studies pointed to an inadequate LH surge as a cause of LUF, which is because of inadequate estrogen activity (Begum, Quadir, Begum, Begum, & Begum, 2006; Sonntag & Ludwig, 2012). Clomiphene citrate has an estrogenic structure and downregulates estrogen receptors in the hypothalamus. It seems that a central antiestrogenic effect and a longer half–life (two weeks) of clomiphene citrate leads to unexpulsion of the oocyte, but letrozole has a shorter half-life (45 hours) and does not reduce estrogen receptors. (M. R. Begum et al., 2009; Begum et al., 2006; Sonntag & Ludwig, 2012). Some researchers pointed out that preovulatory HCG administration is a successful treatment for LUF in ovulatory dysfunction (Coetsier & Dhont, 1996; Vlahos et al., 2005). It is unclear why an unruptured follicle occurs in stimulated cycles despite the administration of HCG. It seems likely that other factors are involved.

In the current study, complete LUF in follicle size 18–19.9 mm, and 20–21.9 mm was lower compared to ≥22 mm. It means that the injection of HCG in preovulatory follicles larger than 22 mm in size leads to more unruptured follicles.

In contrast to our findings, T. Coestier et al. (1996) reported that there is a strong correlation between the follicle size at the day of HCG administration and the probability of ruptured follicles (Coetsier & Dhont, 1996), and that there is a negative relationship between LUF and follicle size in patients with partial LUF (Sonntag & Ludwig, 2012).

Consistent with our study, J. Cuervo-Arango et al. showed that the delayed injection of HCG in mares (in follicle diameter >40 mm) was associated with higher HAF (hemorrhagic anovulatory follicles), because of a spontaneous LH surge before HCG (Cuervo-Arango & Newcombe, 2012). Other studies also pointed out that the ovulation rate was higher in follicles larger than 20 mm at the time of HCG administration (Palatnik, Strawn, Szabo, & Robb, 2012; Silverberg et al., 1991).

Several studies examined the effect of follicular size on the pregnancy rate in IUI cycles and reported contradictory results. Some of those studies achieved a higher pregnancy rate in leading follicles that were 18–22 mm in size, and some found a lower rate of pregnancy when follicles were larger than 20 mm (Mosammat Rashida Begum, Jannatul Ferdous, Anowara Begum, & Ehsan Quadir, 2009).

In this study, the midluteal progesterone level in complete LUF patients were lower than the ovulatory ones, and the luteal phase duration was also shorter. In a study conducted by Ghanem and colleagues (2009), the findings reported similar results (M. E. S. Ghanem et al., 2009). T. Coestsier et al. (1996) illustrated that the only difference between the complete LUF group and the partial or ovulatory groups was that the midluteal progesterone was lower in LUF patients (p < 0.05). This is because serum progesterone was strongly correlated with the number of ruptured follicles (Coetsier & Dhont, 1996).

In contrast with our findings that basal FSH was higher in LUF patients, H. Gublan et al. (2006) reported no significant differences in basal LH, FSH, and the midluteal progesterone level between ruptured and non-ruptured follicles (LUF) in patients (Qublan et al., 2006). J. Cuervo-Arango and colleagues showed in their research that elevated early follicular phase LH concentration has been associated with a higher rate of LUF in women (Cuervo-Arango & Newcombe, 2012). This study showed that endometrial thickness during the day HCG was administered was lower in complete LUF versus ovulatory cycles. Another study found that endometrial thickness between the LUF and ovulatory groups was not significantly different, but some research has shown the proliferation of the endometrium occurs more slowly (Wang, Qiao et al., 2008).

### 4.1 Limitations

In the current study, we included women with a normal basal LH and FSH and found a higher FSH in the complete LUF group. One of our limitations in this study was single blind randomization and the fact that patients were informed of their medication, because they should provide the drugs, but the evaluator was blind to the medications. Another limitation was that we could not evaluate other hormonal patterns during the cycle, such as LH, E2, and the progesterone level on the day HCG injection.

## 5. Conclusion

In summary, letrozole with a lower incidence of LUF is more effective than CC for induction of ovulation in IUI cycles. Our study illustrated that larger follicles in diameter of 22 mm or more were associated with a higher rate of LUF. We could not find similar studies about the effect of follicle size at the time of HCG on the occurrence of LUF. We recommend that further studies investigate and focus on the relationship between follicular size and/or full hormonal profiles and LUF.
